# Supplementary material for: Ticagrelor Increases SIRT1 and HES1 mRNA Levels in Peripheral Blood Cells from Patients with Stable Coronary Artery Disease and Chronic Obstructive Pulmonary Disease
Source: Int J Mol Sci. 2020 Feb 25;21(5):1576. doi: 10.3390/ijms21051576 (PMC7084534; doi:10.3390/ijms21051576)
Supplement: Supplementary file 1 [file ijms-21-01576-s001.pdf]

Supplementary Figure S1

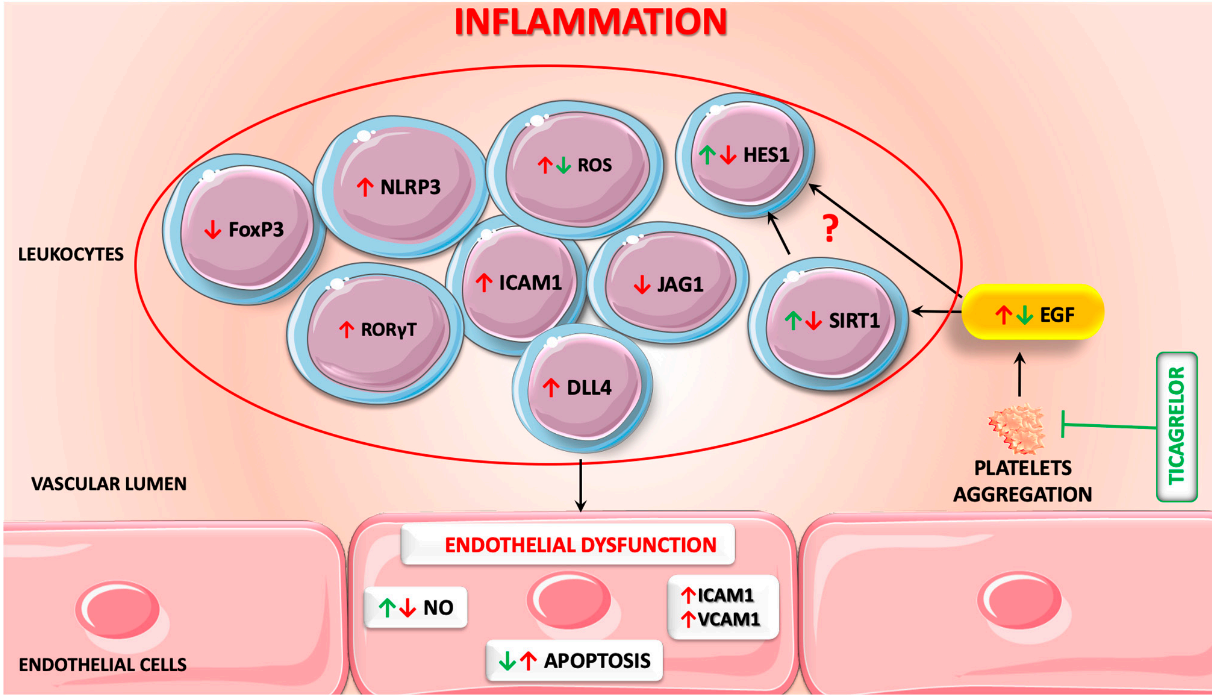

Schematic model representing the effects of ticagrelor on the endothelium of stable coronary artery disease (CAD) /chronic obstructive pulmonary disease (COPD) patients. The inflammatory state (red arrows) is characterized by the following alterations in circulating leukocyte: increased levels of 1) *NLPR3*, 2) *ICAM1*, 3) ROS; decreased levels of *SIRT1* and *HES1*; altered levels of Notch ligand *JAG1* and *DLL4*; altered ratio of Th17/Tregs and their markers *RORγT* and *FoxP3,* respectively. This landscape has been linked to endothelial dysfunction defined by increased apoptosis, increased expression of adhesion proteins (ICAM1, VCAM1) and reduction of NO in endothelial cells. HUVECs treated with serum of patients with stable CAD and COPD following 1-month treatment with ticagrelor show reduced apoptosis and increased NO levels (green arrows), in comparison with HUVECs treated with serum of patients before treatment. Furthermore, in these patients, ticagrelor treatment (green arrows) leads to a decrease of serum levels of EGF and ROS in blood mononuclear cells and to an increase of *HES1* and *SIRT1* mRNA in leukocytes. Based on our analysis and on published data, we propose that part of the effect of ticagrelor on the endothelium could be due to decreased expression of EGF which may lead to the induction of the SIRT1/HES1 axis. Abbreviations: HUVECs: Human Umbilical Vein Endothelial Cells; NO: nitric oxide; ROS: reactive oxygen species; ICAM1: intercellular adhesion molecule 1; VCAM1: vascular cell adhesion molecule 1; FoxP3: forkhead box P3; RORγT: RAR-related orphan receptor gamma; NLRP3: LR pyrin domain containing 3; EGF: epidermal growth factor.

Supplementary Figure S2

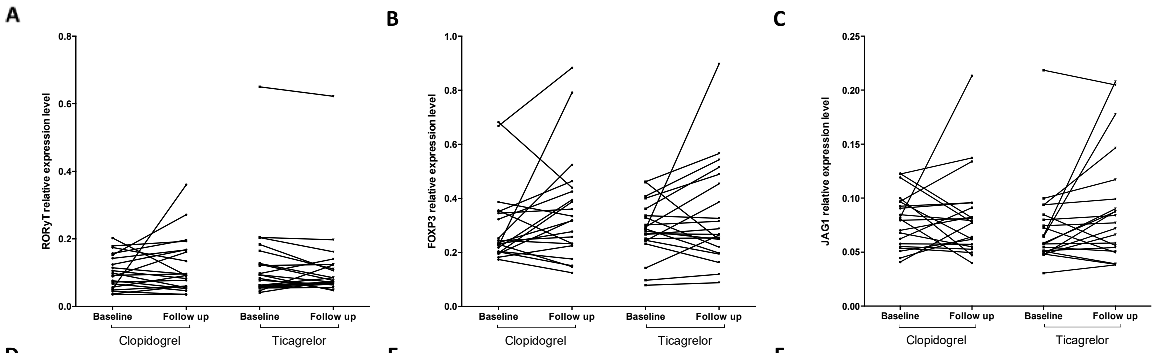

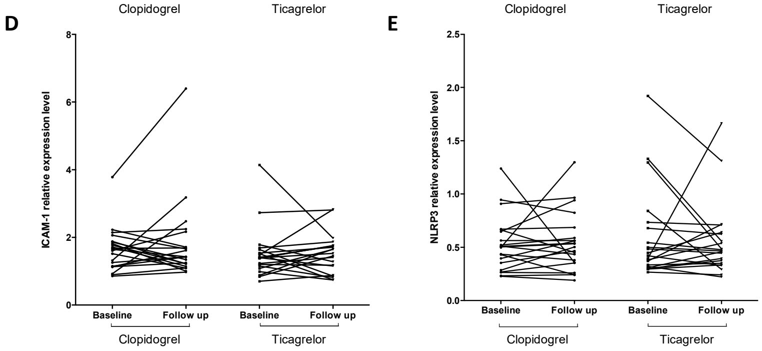

F

	Clopidogrel			Ticagrelor		
	RE	UN	IN	RE	UN	IN
RORγT	6	10	5	9	8	5
FOXP3	5	7	9	5	8	9
JAG1	5	10	6	4	9	9
NRLP3	3	12	6	7	8	7
ICAM1	7	7	7	6	8	8

Before-after graphs showing mRNA expression levels of inflammation- and oxidative stress-related genes at baseline (T0) and follow up (T30). Before-After plot of *RORγt* (A), *FoxP3* (B), *JAG1* (C), *ICAM-1* (D) and *NLRP3* (E) gene expression in peripheral blood cells from patients before and after one month of treatment with clopidogrel or ticagrelor. (F) Number of patients showing reduced (RE, fold change <0.8), unchanged (UN) or increased (IN, fold change > 1.2) levels of each gene expression in peripheral blood cells, following 1-month treatment with clopidogrel or ticagrelor.

Supplementary Figure S3

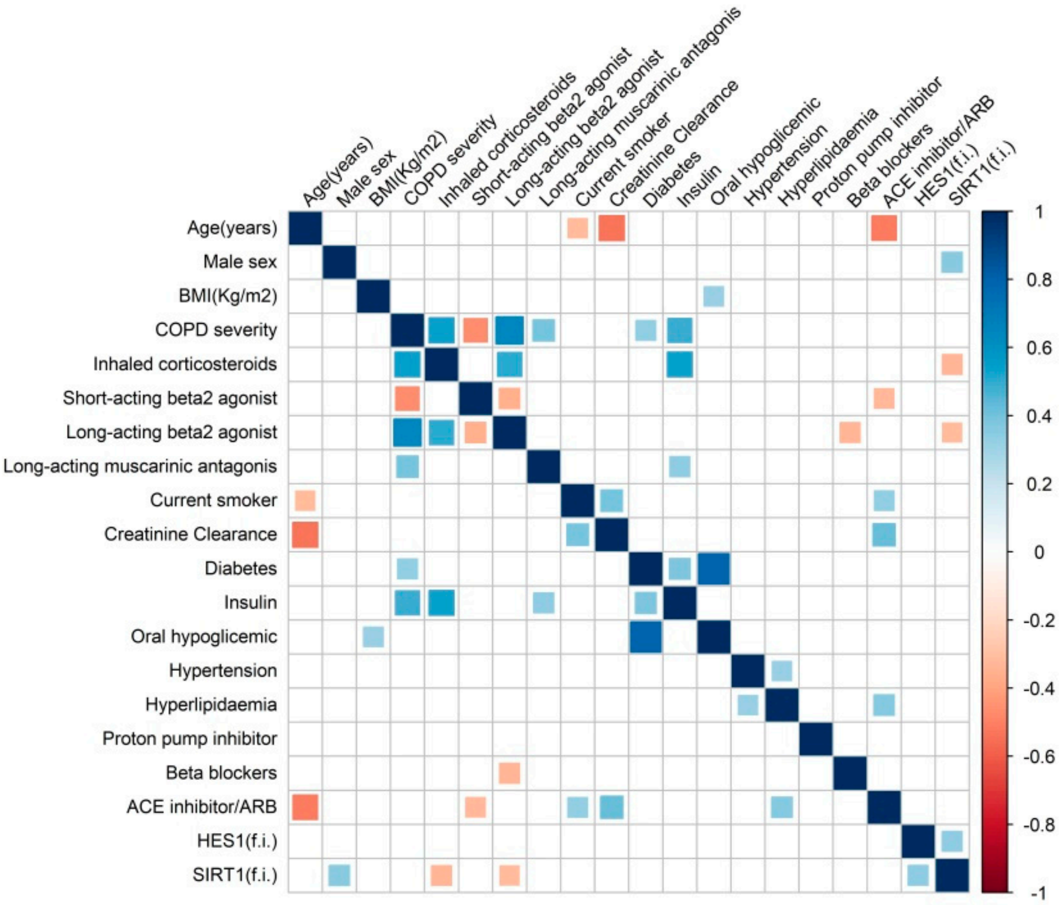

Correlation plot of baseline clinical variables, pharmacological treatment and variation in gene expression. Correlations with $p < 0.05$ were plotted: blue/red color indicates correlation coefficients. BMI: body mass index. COPD: chronic obstructive pulmonary disease. ACE: angiotensin converting enzyme. ARB: angiotensin II receptor blocker. *: pantoprazole was the only proton pump inhibitor administered.

Supplementary Table S1: Primers used for quantitative RT-PCR

	HOMO SAPIENS PRIMER SEQUENCE (5'-3')
**GUSB F**	CCCGCGGTCGTGATGTGGTC
**GUSB R**	GCCGGGAGGGGTCCAAGGAT
**SIRT1 F**	AGTAGGCGGCTTGATGGTAA
**SIRT1 R**	CCTCAGCGCCATGGAAAATG
**NLRP3 F**	CGGGGCCTCTTTTCAGTTCT
**NLRP3 R**	CCCCAACCACAATCTCCGAA
**ICAM-1 F**	AGCTTCGTGTCCTGTATGGC
**ICAM-1 R**	TTTTCTGGCCACGTCCAGTT
**FoxP3 F**	TCACCTACGCCACGCTCAT
**FoxP3 R**	ACTCAGGTTGTGGCGGATGG
**RORyT F**	GAGAAGGACAGGGAGCCAA
**RORyT R**	CCACAGATTTTGCAAGGGA
**JAG1 F**	GACTCATCAGCCGTGTCTCA
**JAG1 R**	TGGGGAACACTCACACTCAA
**DLL4 F**	GCGAGAAGAAAGTGGACAGG
**DLL4 R**	ATTCTCCAGGTCATGGCAAG
**HES1 F**	CGGACATTCTGGAAATGACA
**HES1 R**	CATTGATCTGGGTCATGCAG
